# The assessment of health system responsiveness from the viewpoints of COVID-19 patients

**DOI:** 10.1186/s12913-023-09806-9

**Published:** 2023-08-24

**Authors:** Ehsan Teymori, Seyed Saeed Tabatabaee, Saeed Akhlaghi, Azam Delavarinejad, Fatemeh Kokabisaghi

**Affiliations:** 1 Student Research Committee, School of Health, Mashhad University of Medical Sciences, Mashhad, Iran; 2 Department of Health Economics and Management Sciences, School of Health, Mashhad University of Medical Sciences, Mashhad, Iran; 3 Biostatistics Department, School of Health, Mashhad University of Medical Sciences, Mashhad, Iran

**Keywords:** Health system responsiveness, Patient rights, Covid-19 pandemic, Iran

## Abstract

**Background:**

Pandemics such as Corona are currently major health concerns worldwide. Health system responsiveness to the medical and non-medical needs of patients during pandemics is essential. This study aimed to examine hospitals’ responsiveness to Corona patients.

**Methods:**

This descriptive and analytical research had a cross-sectional design. The study population included Corona patients discharged from 17 public hospitals of Mashhad University of Medical Sciences, Iran, in the spring of 2021. WHO questionnaire for health system responsiveness was used to collect data. 413 patients participated in the study who were selected by random classified sampling. To analyze the data, descriptive statistics, including frequency, and deviation, and to examine the relationship between variables, Kruskal-Wallis and Mann-Whitney tests were used.

**Results:**

In this study, one-third participants were in the age range of 31 to 40 (32.6%). The ability of 277 (70.5%) participants to pay treatment costs was very low, and low. 380 (96.7%) of the respondents had basic health insurance and 101 (25.7%) had supplementary insurance. In general, respondents evaluated the responsiveness of hospitals as 75.6. The highest score was related to confidentiality, and the lowest to prompt attention. There was no significant relationship between the total response score with demographic information.

**Conclusion:**

The responsiveness of studied hospitals to Corona patients was adequate. However, there was dissatisfaction with the lack of timely treatment and medication. Moreover, the most important dimension of responsiveness was dignity. Healthcare providers need to pay attention to different aspects of responsiveness and improving the quality of and access to health services during pandemics and disasters.

## Background

The Covid-19 pandemic has created extraordinary challenges for the health systems of almost all countries around the world. It profoundly impacted the life of people, society and the economy. Health systems around the world were not prepared fully to face a pandemic, despite having plans to deal with other infectious diseases such as influenza. In low and middle-income countries, the lack of human resources, infrastructure, and equipment to deal with the pandemic was more evident [1]. More than two years after the Covid-19 outbreak, health systems learned that there should be plans for managing the next probable crisis. Moreover, special attention needs to be paid to key aspects of health systems such as system responsiveness.

To evaluate the performance of health systems, the World Health Organization introduced a framework consisting of health, responsiveness, and fairness in financing [2]. “Responsiveness in the context of a system can be defined as the outcome that can be achieved when institutions and institutional relationships are designed in such a way that they are consistent and respond appropriately to the universally legitimate expectations of individuals.” [3] In fact, responsiveness refers to the response to people’s rational expectations regarding the non-medical aspects of the health system. Responsiveness is also associated with patient satisfaction, usually related to treatment advice, real-time action, better understanding and retention of medical information [4].

Measuring responsiveness makes it possible to understand the different characteristics of the health system and, as a main criterion, to take into account how the citizens evaluate care and respond to it [5]. In recent years, the importance of patient communication skills and attention to the patient in treatment and care has been increasingly emphasized [6]. The relationship between the therapist and the patient goes beyond a contract and becomes a relationship based on mutual trust and heartfelt belief. Patients, particularly in the public sector, are faced with employees who do not have enough motivation and proper training, and long waiting lists and lack of timely treatment [7]. Health system evaluation provides decision-makers with timely and relevant information regarding health system performance that is needed to move towards national goals and policies [8].

During the Corona pandemic, patients occupied almost all public and private healthcare facilities. Healthcare personnel, particularly nurses, feared the unknown disease and insufficient protection [9]. On the other hand, patients suffered shortages of medicines, equipment, and facilities. The situation was worse for the uninsured ones who did not have the resources to pay for services [10]. In addition, patients and healthcare providers experienced stigma. Some healthcare personnel was reluctant to provide services to Corona-infected patients [11]. Denial to treat Corona patients is a violation of the right to health. Considering the importance of health system responsiveness in pandemics, this study aimed to investigate it during the outbreak of Coronavirus. The results will help to identify the strengths and weaknesses of current health system responsiveness programs.

## Methods

This descriptive-analytical research has a cross-sectional design. The study population was Corona patients discharged from 17 public hospitals affiliated with Mashhad University of Medical Sciences, Iran. These hospitals have a total of 3907 beds. Six hospitals are regional and specialized and 11 general hospitals. During Corona outbreak, they were converted to designated hospitals for COVID-19 patients.

The methods of data collection were questionnaires and interviews. The questionnaire designed by WHO for evaluating health system responsiveness was used in this study. By considering the study by Hoffmann et al. (with a response rate of 68% in several European countries) [12] and taking into account the possibility of dropping samples (10%), with 95% confidence and 0.045 accuracy, the sample size was determined using Cochrane formula to be 393.

1 $$n = \frac{{\frac{{{Z^2}pq}}{{{d^2}}}}}{{1 + \frac{1}{N}\left( {\frac{{{Z^2}pq}}{{{d^2}}} - 1} \right)}}$$

Study participants were selected by random classified sampling. First of all, the number of samples for each hospital was determined based on the number of their beds. Then, the study participants were randomly selected from the patients discharged from each studied hospital in March 2022. To fill out the questionnaires, the researchers contacted study participants by phone and administrated interviews. The questionnaire had three parts, including the patient’s demographic information, health system responsiveness dimensions (dignity, autonomy, confidentiality, prompt attention, social support, quality of basic amenities, and choices of providers), and two open-ended questions; “which dimension of responsiveness is more important to you?” and “how do you think health system responsiveness can be improved?”

A five-point Likert scale was used to score the answers to the questionnaire (0–5). The average score of each area was calculated between 0 and 4 so that if the response average was less than 1.33, it was considered poor, between 1.34 and 2.66 as average and above 2.67 as appropriate. From the average scores of all dimensions, the total response score was determined. To analyze the data of the first and second parts of the questionnaire, SPSS version 20 was used. Descriptive statistics, including frequency, mean, and standard deviation according to the type of variable, were calculated. Also, Mann-Whitney and Kruskal-Wallis tests were used to check the relationships between variables, including different age groups, and gender. The collected data for the third part was coded and classified using MAXQDA version 10.

## Results

Most participants were in the age range of 31 to 40 years (32.6%). 213 (54.2%) participants were employed and 166 (42.2%) had undergraduate education. 151 (38.4%) participants stated that their income level was very low, and 126 (32.1%) evaluated it as low compared to the costs of treatment. 380 (96.7%) participants had basic medical insurance, and 101 (25.7%) had supplementary medical insurance. Table 1 shows the demographic characteristics of the studied patients.

**Table 1:** Demographic information of study participants

	Variable	Values	percentage/number
1	Age	19–30	(10.2)40
		31–40	(32.6)128
		41–50	(26)102
		51–60	(31)122
		Older than 60	(0.3)1
2	Gender	Female	(50.6)199
		Male	(49.9)194
3	Working	Yes	(54.2)213
		No	(45.5)179
4	Education	Primary school	(42.2)166
		High school	(32.8)129
		Bachelor	(19.1)75
		Master and higher	(5.3)21
5	Income	Very low	(38.4)151
		Low	(35.9)141
		Average	(3.8)15
		High	(11.5)45
		Very high	(0.1)4
		Prefer not to answer	(9.4)37
6	Costs of treatment compared to income	Very low	(6.1)24
		Low	(32.1)126
		Average	(12.5)49
		High	(24.7)97
		Very high	(0.5)2
		Prefer not to answer	(6.1)24
7	Basic health insurance	Yes	(96.7)380
		No	(3.3)13
8	Supplementary health insurance	Yes	(25.7)101
		No	(74.3)292

The study participants gave the lowest score to the dimension “prompt attention” (3.68) and the highest score to “confidentiality” (4.38). According to them, the general health responsiveness score was 3.78. The scores of all dimensions are shown in Table 2.

**Table 2:** The health system responsiveness situation in Corona hospitals

	Dimensions	Average score	Standard deviation
1	Dignity	4.24	4.71
2	Autonomy	4.09	4.54
3	Confidentiality	4.38	3.77
4	Choice of provider	4.05	3.36
5	prompt attention	3.68	4.99
6	basic amenities quality of	4.1	4.8
7	social support	4.07	3.98

Based on the results of the Mann-Whitney and Kruskal-Wallis tests, there was no significant relationship between the total responsiveness score and the demographic variables examined except for supplementary insurance. Among the seven investigated dimensions, the dimensions of dignity, confidentiality, autonomy and supplementary insurance were found to have a significant relationship. Also, there was a significant relationship between income and dimensions of confidentiality and autonomy (p = 0/007) (Table 3).

**Table 3:** The relationship between variables and health responsiveness dimensions

dimension	gender	Age	Supplementary insurance	education	employment	income
Dignity	0.451	0.337	0.001	0.16	0.72	0.024
Autonomy	0.81	0.162	0.016	0.347	0.593	0.334
Confidentiality	0.883	0.352	0.259	0.246	0.523	0.033
Choice of provider	0.619	0.488	0.149	0.431	0.48	0.662
Prompt attention	0.489	0.239	0.001	0.356	0.328	0.007
Quality of basic amenities	0.432	0.385	0.003	0.424	0.357	0.003
Social support	0.455	0.478	0.145	0.31	0.423	0.028
General	0.881	0.196	0.03	0.597	0.588	0.053

The most important dimension of health system responsiveness for the participants was dignity (37.2%). Access to services (28.2) and the basic amenities (20.2) were ranked second and third from the patients’ point of view, respectively (Table 4).

**Table 4:** The importance of health system responsiveness according to the study participants

	Dimension	number (percentage)
1	Dignity	(37.2)146
2	Prompt attention	(28.2)111
3	Quality of basic amenities	(20.2)40
4	Choice of provider	(3.1)12
5	Confidentiality	(1)4
6	Autonomy	(0.3)1
7	social support	(1)4
8	All dimensions	(20.1)79

Most participants believed that prompt attention can improve responsiveness score. (n = 63). Patients did not suggest anything about the choice of provider. The suggestions can be seen in Table 5.

**Table 5:** Study participants’ suggestions to improve health system responsiveness

Dimensions	The suggestion	Number of patients suggested	sum
Basic amenities	Improving the quality and variety of food	8	18
	Improving indoor and outdoor facilities such as garden, parking places, waiting rooms, and air-conditioning and the cleanliness of wards	10	
Prompt attention	Prompt attention	24	63
	Health products and medicines	16	
	Healthcare equipment	12	
	Shortening waiting lists	7	
	Training healthcare personnel	4	
Dignity	Equal treatment	2	17
	Treating everyone in their care with dignity and humanity	15	
Social support	Assigning personnel to help patients to eat and take a shower	2	9
	Mental and spiritual healthcare	7	
Autonomy	Educating patients about the disease and side effects of medicines	5	5
Privacy and confidentiality	Providing healthcare by providers of the same gender as patients	6	6

## Discussion

Health system responsiveness was satisfactory in the studied hospitals. The study by Fazaeli et al. (2016), which investigated the health system responsiveness in outpatient care facilities in Mashhad [13], is in line with the results of our study. Hoffmann et al. study showed that on average, two-thirds of the people evaluated all areas of health system responsiveness as good and very good [12]. According to Zarei et al., health system responsiveness was at moderate level in Tehran. Choice of provider, autonomy and prompt attention received the lowest scores in their study [13]. In primary health care facilities in Tanzania, health system responsiveness was poor as the study by Kapologwe et al. indicated [14].

It was challenging for countries to manage pandemic of coronavirus disease. Some countries considered the most prepared for a pandemic, such as the United States and the United Kingdom, have performed the worst [15]. Bong et al. [16] predicted that the COVID-19 pandemic would negatively impact healthcare due to the severe shortages already experienced. The shortage of health workers in American and European countries has worsened due to the pandemic. Globally, the COVID-19 pandemic has reduced the number of health workers available to keep health systems functioning [17, 18]. For example, the lack of human resources in Nigeria has fueled the challenges of the country’s health system [19, 20]. In countries where many people live in poor economic conditions, it is much more difficult to properly respond to it, as these populations have to choose between being quarantined and receiving treatment or going to work to support living expenses [21, 22].

The most important dimensions of responsiveness from the patient’s point of view in the present study were, respectively, dignity and prompt attention, which are consistent with the results of the study of Sao Jose (2016), Chan (2015) and Baharvandi (2019) [23–25]. Because of the unknown nature of the disease and the fear, the patients wished prompt attention. Moreover, It can create stigma against people and communities, fear and anxiety that can lead to social isolation and prejudice [11]. This situation undermines the dignity of people.

The best performance in terms of responsiveness was related to the dimension of confidentiality, and the worst performance was related to the dimension of access to services, that are in line with the results of the study by Mohammadi and Kamali (2015) [26]. In their study, the best performance was in the confidentiality dimension, and the worst was related to the autonomy. Confidentiality was important for the patients because of the fear of stigma and discrimination based on the disease. Prompt attention is very related to access to health services, products and facilities particularly in crisis and pandemics. The weak health system in Nigeria lacked adequate medical products and technologies needed to provide care for patients with severe respiratory failure [17]. In the current research, the dimension of prompt attention and access to healthcare received the lowest score. As a new disease, the COVID-19 pandemic has had a devastating impact on service delivery. Before the pandemic, health inequalities were observed in some countries and it was predicted that the inequality would worsen during the epidemic [27]. The patients who needed surgery were denied access to it due to assigning all personnel and other resources to provide care for patients with Covid-19 [22].

The World Health Organization has defined the right to health as the right to good quality care and treatment, fair access to healthcare and services, confidentiality of information and informed consent and autonomy [28]. According to Jia et al.‘s (2020), respecting patients’ rights is one of the ethical challenges of health systems during pandemics [29]. In Malekzadeh et al.‘s study, service providers know it is necessary to respect the fundamental rights of patients. But due to the large number of hospitalized patients and the possibility of the disease spreading to those around the patients, the medical staff faces an ethical challenge that the confidentiality of the patient must be respected or safety of the patient’s family members and relatives [30]. Patients are entitled to decide whether and to whom their personal health information is disclosed. Giving them information on the importance of their cooperation in preserving the health of others might be helpful.

Respecting patient autonomy and participation in treatment is a key component in providing care, which requires the cooperation of service providers and enhancing the patient’s ability to understand the best decision over time [31]. According to the results of Pelto-Piri’s study, the medical staff’s information about Corona disease was inadequate. As a result, they were not able to provide sufficient information to the patients. If the patient’s decision were not to accept the treatment and comply with the principles of quarantine, the medical staff would face a challenge. In such situations, the traditional care approach based on the provider’s decision could be more practical [32]. In the present study, no challenge was reported by patients regarding autonomy and making informed decisions. The cause of this issue can be related to the nature of Covid-19 disease, as the patient left the choice of treatment to the doctor. Also, most patients did not know about their rights, such as choosing a doctor and nurse, and a smaller number of patients had chosen their doctor. In a system with paternalistic culture, people usually leave making the decisions to healthcare providers. The preferences of patients are not asked in such systems.

In this study, there was no significant relationship between the total responsiveness score with any of the demographic variables (gender, age, level of education, and occupation), which is in line with the results of Keyvanloet al. [33]. But it is not consistent with the study of Fazaeli et al., which aimed to measure the quality of non-medical services in Mashhad teaching hospitals [34]. In the study of Fazaeli, a significant relationship was found between income, gender, and the level of education with responsiveness. In the study of Rashidian (2011) aimed at determining health system’s responsiveness and the factors affecting it in Tehran [35], a significant relationship was observed between gender and responsiveness. In the present study, a significant relationship was found between the dimensions of health system responsiveness, including dignity, confidentiality, and having supplementary insurance. More studies are needed to explain this relationship.

## Conclusion

The Covid-19 pandemic has had a major impact on health systems around the world. In this study, health system responsiveness to Corona patients received an acceptable score. Patients believed that dignity was the most important dimension of responsiveness. They were satisfied with confidentiality, and believed that easy access to good quality medical services and equipment should be improved. In the studied hospitals, there was dissatisfaction about long waiting lists and shortage of medicine. This problem was related to the nature of the pandemic and the unpreparedness of the health system.

A variety of factors in different cultures and societies might influence people’s perception of health system responsiveness. Hospital managers and healthcare providers need to pay attention to different aspects of responsiveness and improving the quality of and access to health services. Facilitating effective information flow between the health system and the public is a key component of responsiveness. People should have the opportunity to participate in health system decisions. Good governance is essential to ensuring respect for human rights. Training health system personnel on people’s rights would be helpful to enhance responsiveness. Further studies on how to promote patients’ rights and facilitate their participation in health system decision making are necessary.

## Study strengths and limitations

This study had several strengths; it included a large number of hospitals in the second most populous city of Iran during the second wave of Corona disease, and the results can be generalized confidently. Moreover, because of the nature of the disease and the fear of disease transmission, we decided to collect data via phone calls. More than 1200 calls were made in the study period. The results can help understanding of the health systems responsiveness, and guide actions to further strengthen health systems responsiveness.

This study did not include the patients who passed away during hospitalization or one month after discharge. Also, children, because of their different mentality from adults, were excluded. Researchers believe that children need to be studied with different considerations for age.

## Data Availability

The datasets used and analyzed during the current study are available from the corresponding author upon reasonable request.
